# Remifentanil infusion as a modality for opioid-based anaesthesia in paediatric practice

**DOI:** 10.4103/0019-5049.68375

**Published:** 2010

**Authors:** Ahmed Mostafa Abdel Hamid, Ashraf Fawzy Abo Shady, Ehab S Abdel Azeem

**Affiliations:** Department of Anaesthesia and ICU, Benha Faculty of Medicine, Benha University, Egypt

**Keywords:** Opioid based, paediatric, remifentanil

## Abstract

This study was designed to compare the intra-operative and post-operative analgesic requirements and side effects of using fentanyl infusion versus remifentanil infusion during short-duration surgical procedures in children. The study comprised of 40 children randomly allocated into two equal groups: fentanyl (F-group) or remifentanil (R-group). Both were administered a continuous intravenous (i.v.) infusion. Anaesthetic recovery was assessed using the Brussels sedation scale every 5 min from the time of entry till discharge from recovery room. Post-operative analgesia was assessed throughout the first three post-operative (PO) hours using observational pain–discomfort scale (OPS) and adverse events were recorded. Haemodynamic variables showed a non-significant difference between both the groups. Patients who received remifentanil showed significantly shorter time to spontaneous respiration, eye opening, extubation and verbalization compared to those who received fentanyl. Discharge time was significantly shorter in R-group, and 18 patients fulfilled criteria for recovery-room discharge at ≤25 min with a significant difference in favour of remifentanil. Fentanyl provided significantly better PO analgesia than remifentanil and children in F-group showed a significantly lower mean cumulative OPS record than those in R-group; however, the number of patients requiring rescue analgesia did not show a significant difference between both the groups. Two cases in F-group and one in R-group had bradycardia, one case in R-group had mild hypotension and PO vomiting had occurred in three patients in the F-group and two patients in the R-group. In conclusion, remifentanil is appropriate for opioid-based anaesthesia for paediatric patients as it provides haemodynamic stability and rapid recovery with minimal post-operative side effects.

## INTRODUCTION

Remifentanil is a µ-opioid receptor agonist with an analgesic potency similar to that of fentanyl.[1] Remifentanil is an analogue of fentanyl (4-piperi-dyl anilide) with a methyl-ester group that allows the molecule to be hydrolysed by non-specific tissue and plasma esterases.[2] Rapid biotransformation to minimally active metabolites should be associated with a short duration of action with no accumulation of effect on repeated dosing or with continuous infusion.[3] These pharmacokinetic properties could explain the rapid onset and short duration of action of remifentanil.[4]

Remifentanil has a rapid onset, rapid offset, small volume of distribution, rapid clearance, and a short elimination half-life,[5] so, it may be a useful anaesthetic for paediatric outpatient surgery.[6]

Opioids are often used in combination with propofol. The combination of these two drugs may be particularly useful for procedures of short duration.[7]

Combining a hypnotic and an analgesic to produce sedation, analgesia and surgical immobility is more common than administration of a volatile anaesthetic alone, and response surface analyses demonstrate a synergistic interaction between remifentanil and sevoflurane for sedation and all analgesic endpoints.[8]

This study was designed to compare the outcome of using fentanyl infusion versus remifentanil infusion in conjunction with a volatile anaesthetic during short-duration surgical procedures in children.

## METHODS

This study comprised 40 children, aged 3.5–8 years, assigned to undergo lower abdominal hernia repair procedures under general anaesthesia. After obtaining informed parental consent, children were randomly allocated into two equal groups (*n*=20 patients) according to opioid to be used; either fentanyl (F-group) or remifentanil (R-group). Patients with contraindication to the use of either of the study drugs or inhalational anaesthesia and patients with respiratory pathology were excluded from the study. Single blinding technique was used. Standard monitoring included electrocardiogram (ECG), pulse oximetry (SpO_2_), non-invasive blood pressure (NIBP) and end tidal carbon dioxide (ETCO_2_). All patients were premedicated using oral midazolam 0.15 mg/kg, and anaesthesia was induced with sevoflurane 6% in oxygen 6 L/min via facemask. After an IV cannula was inserted, atracurium 0.3–0.5 mg/kg was administered to facilitate tracheal intubation and provide muscle relaxation. Controlled ventilation via closed circuit was used. The ventilation parameters are oxygen 100%, tidal volume 7–10 mL/kg, respiratory rate 16–20/min and I:E (Inspiratory to Expiratory ratio) ratio 1:2. Throughout the operative procedure, sevoflurane concentration was 3% and ETCO_2_ was maintained at 35–45 mmHg. Before surgical manipulation, ondansetron (100 µg/kg) and dexamethasone (0.25–0.5 mg/kg) were administered i.v. to prevent post-operative (PO) nausea and vomiting (PONV)

Fentanyl was initially given as a continuous i.v. infusion at 5 µg/kg/min. After endotracheal intubation, fentanyl infusion was reduced to 2.5 µg/kg/min. Remifentanil was initially given as a continuous i.v. infusion at 0.5 µg/kg/min and after endotracheal intubation, remifentanil infusion was reduced to 0.25 µg/kg/min. The infusion rates were later adjusted as required to treat light anaesthesia responses classified by autonomic responses (lacrimation, sweating, increase of blood pressure or heart rate), or anticipated changes in magnitude on surgical stimulation.

Light anaesthesia responses were recorded at the time of induction, one and five minutes after intubation, at time of skin incision and closure, and five minutes after transfer to the recovery room. Incidence of adverse events were monitored throughout the study period included: 1) hypotension (≥20% decrease compared with baseline blood pressure), 2) bradycardia defined as heart rates <80 beats per minute for at least 1 min. Hypotension was treated by infusion of lactated Ringer’s solution, and for bradycardia atropine sulphate (0.01 mg/kg) was administered.

Ten minutes before anticipated end of surgery, the study drugs infusion rate was decreased to 0.5 µg/kg/min in F-group and to 0.05 µg/kg/min in the R-group. At the end of surgery, neuromuscular block was reversed with neostigmine and atropine and the infusion of drugs was stopped. Anaesthetic recovery was assessed using the Brussels sedation scale, graded as unarousable (level 1), responds to painful not auditory stimuli (level 2), responds to auditory stimuli (level 3), awake, calm, able to follow commands and had motor function unchanged from their pre-operative evaluation (level 4) and agitated (level 5). Patients were considered to have normal recovery on approaching level 4 on Brussels sedation scale. Recovery score was recorded every five minutes from the time of entry till discharge from recovery room. The recovery-room discharge time was defined as the time elapsed from cessation of the anaesthesia till reaching level 4 on Brussels sedation scale and before being agitated and required analgesia.

Post-operative analgesia was assessed throughout the first three PO hours using observational pain–discomfort scale (OPS), which assessed behavioural objective parameters, namely crying, facial expression, position of tarso, position of the legs, and motor restlessness. Each variable was scored on a three-point scale (1 = none, 2 = moderate, and 3 = severe) to give a cumulative score of 5–15 to estimate the quality of analgesia (5 = excellent, 15 = ineffective). Adverse events like hypotension, bradycardia, hypoxia, and dysarrythymias and the occurrence of PO vomiting were recorded.

Statistical analysis was conducted using the SPSS (Version 10, 2002) for Windows statistical package, using *t*-test for independent sample and Chi-square test.

*P* value <0.05 was considered statistically significant with the power of the study being 90%.

## RESULTS

The study included 40 patients with mean age of 5.4 ± 1.4; range: 3.5–8 years and mean weight of 18.9 ± 2.8; range: 15–24 kg. There were 35 patients ASA grade I and 5 patients ASA grade II. All patients completed the study with a non-significant (*P*>0.05) difference between both groups in the terms of age, body weight, gender distribution, and ASA classification. The duration of surgery and anaesthesia was 55.2 ± 11.4 and 64.6 ± 13.3 min, respectively. Mean dose of ondansetron and dexamethasone used was 2 ± 0.3 and 4.9 ± 0.8 mg, respectively. Also, both groups were comparable with a non-significant difference as regards to duration of surgery and anaesthesia and dose usage of ondansetron and dexamethasone [Table 1].

**Table 1:** Patient demographics and operative data

Data	F-group (n=20)	R-group (n=20)	*P*
Age (years)	5.7±1.3	5.3±1.5	0.4
Sex; M:F	11:9	13:7	0.7
Weight (Kg)	19.1±2.7	18.6±3	0.6
ASA; I:II	17:3	18:2	0.6
Duration of surgery (min)	54.9±12.4	57.2±10.7	0.5
Duration of anaesthesia (min)	64.2±14.5	67±12.5	0.5
Dose of ondansteron (mg)	2±0.3	1.98±0.3	0.8
Dose of dexamethasone (mg)	4.9±0.8	5±0.8	0.7

**Table 2:** Haemodynamic responses detected in studied groups

Data		F-group (n=20)	R-group (n=20)	*P*
SBP (mmHg)	Baseline	78±4.6	78.7±4	0.6
	Induction	74.8±4.4	73.4±4.3	0.3
	1-min after intubation	82.2±6.9	81.5±6.2	0.7
	5-min after intubation	78.7±5.2	77.3±4.6	0.4
	Skin incision	67.3±4.5	68±5.2	0.7
	Skin closure	76.3±4.1	75.3±4.8	0.5
	At discharge from recovery room	82.4±7.5	80.7±4.8	0.4
DBP (mmHg)	Baseline	52.3±3.2	51.3±4.7	0.4
	Induction	50±2.4	49.3±5.6	0.6
	1-min after intubation	51.3±4	54.3±6	0.8
	5-min after intubation	51.9±2.4	53.8±5.6	0.2
	Skin incision	48±3.3	47±4.1	0.4
	Skin closure	49.3±4.2	50±1.5	0.5
	At discharge from recovery room	48.7±3.5	50.7±1.8	0.02*
HR (bpm)	Baseline	131.9±5	128.1±3.7	0.009*
	Induction	133.1±5.6	132.3±6.9	0.7
	1-min after intubation	137.6±5.9	136.7±5.9	0.6
	5-min after intubation	134.9±8.1	133.3±5.6	0.5
	Skin incision	131±7.6	126.1±3.6	0.01*
	Skin closure	131.7±7.1	129.3±3.7	0.2
	At discharge from recovery room	133.2±5	130±3.7	0.02*

* means significant

**Figure 1:**
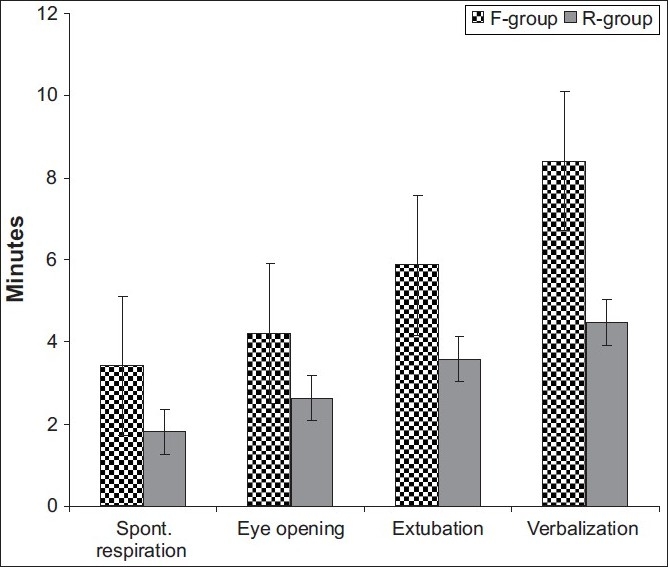
Mean (±SD) emergence time after discontinuation of anaesthetic

Haemodynamic variables recorded throughout the study period showed a non-significant difference (*P*>0.05) in response to the use of either remifentanil or fentanyl despite the responses detected at intubation, skin closure, and light anaesthesia being more frequent in fentanyl group [Table 2]. Patients who received remifentanil showed superior emergence of anaesthesia compared to those who received fentanyl as shown by significantly shorter time (*P*<0.05) to respire spontaneously, to eye opening, to extubation and to verbalization [Table 3, Figure 1].

**Table 3:** Mean (±SD) emergence time after discontinuation of anaesthetic

Time (min) to	F-group (n=20)	R-group (n=20)	*P*
Spontaneous respiration	3.4±1.92	1.8±0.82	0.004*
Eye opening	4.2±1.9	2.63±0.44	0.014*
Extubation	5.87±1.81	3.57±0.53	0.012*
Verbalization	8.4±1.64	4.47±0.67	0.010*

* means significant

**Figure 2:**
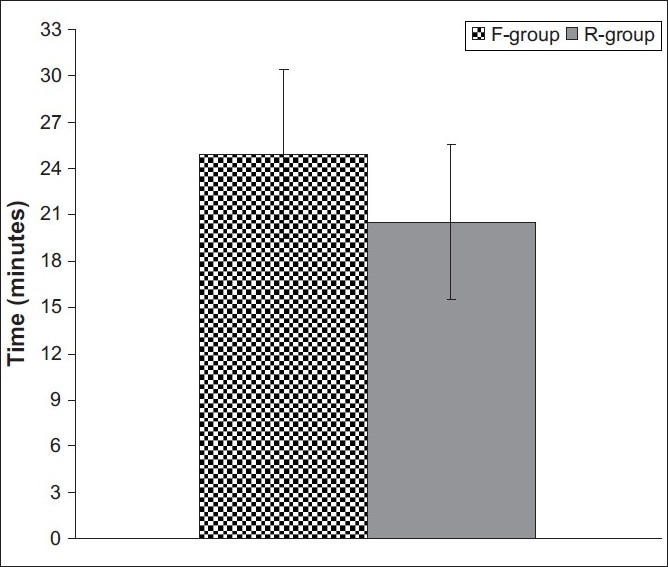
Mean time (±SD) till discharge from the recovery room estimated in both groups

**Figure 3:**
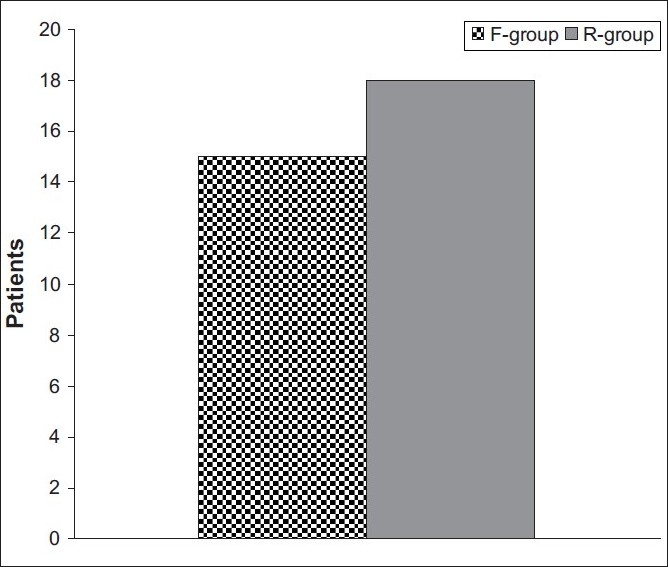
Number of patients ready for discharge from the recovery room at 25 minutes in both groups

Time to qualify for recovery-room discharge was significantly shorter (Z = 2.101, *P* = 0.036) in patients who received remifentanil, (20.5 ± 5 min) compared to those who received fentanyl (24.9 ± 5.5 min) [Figure 2]. Furthermore, 18 patients (90%) fulfilled criteria for recovery-room discharge at ≤25 min in case of remifentanil group, while 15 patients (75%) who received fentanyl were ready to discharge at ≤25 min, with a significant difference (χ^2^ = 5.733, *P* = 0.01) in favour of remifentanil [Figure 3].

**Figure 4:**
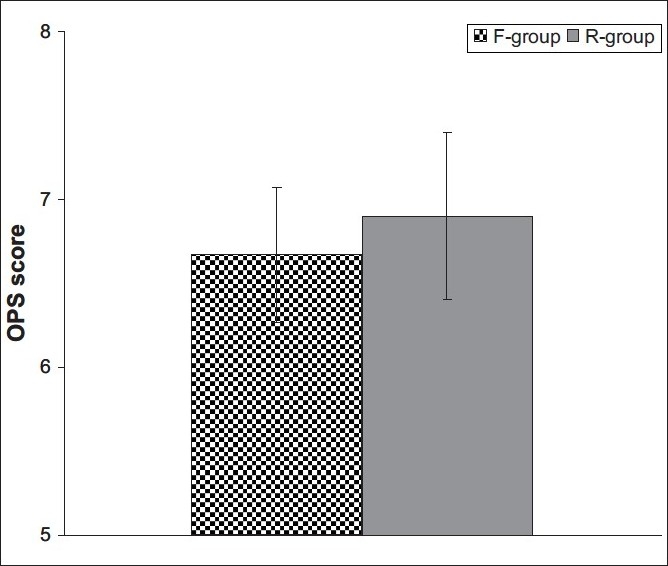
Mean (±SD) OPS score determined in both groups throughout the first 3 postoperative hours

**Figure 5:**
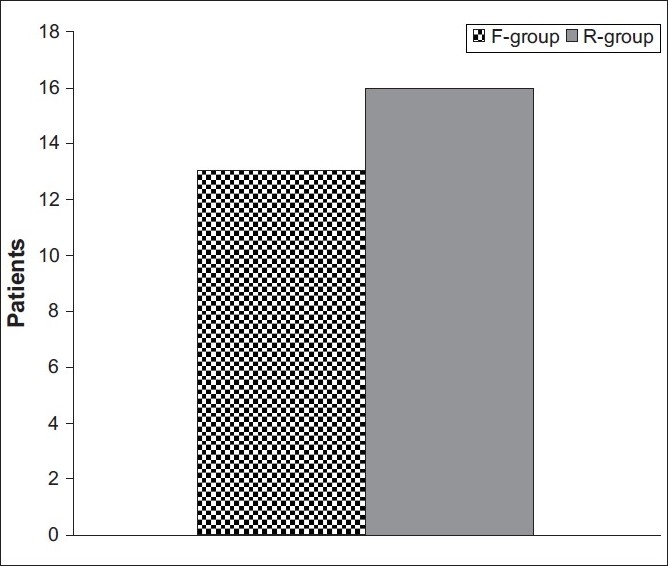
Patients’ distribution according to request of postoperative analgesia

Fentanyl provided significantly better PO analgesia than remifentanil; children administered fentanyl showed a mean cumulative OPS record throughout the first three PO hours, which was significantly (Z = 2.201, *P* = 0.028) lower than that recorded for children who received remifentanil (6.67 ± 0.4 vs. 6.9 ± 0.5, respectively) [Figure 4]. However, number of patients required rescue analgesia despite increase in both groups did not show a significant difference (χ^2^ = 0.094, *P* > 0.05) between children who received fentanyl (13 patients) or remifentanil (16 patients) [Figure 5].

No cases of dysrhythmia were detected; however, two cases in fentanyl group, while one in remifentanil group had bradycardia that required intravenous injection of atropine sulphate. Only one case of mild hypotension was detected in remifentanil and required fluid therapy for adjustment of blood pressure. Hypoxia (decreased O_2_ saturation less than 90%) was encountered in only one case in fentanyl group. Post-operative vomiting had occurred in five patients, three in fentanyl group and two patients in remifentanil group [Table 4]. Despite the apparent increased incidence of adverse events in fentanyl group, the difference did not reach the significance level (χ^2^ =0.012, *P*>0.05).

**Table 4:** Adverse events occurring intra- and post-operatively

Data	F-group (n=20)	R-group (n=20)	*P*
Hypotension	0	1 (5)	
Bradycardia	2 (10)	1 (5)	
Hypoxia	1 (5)	0	0.9
Dysrhythmia	0	0	
Vomiting	3 (15)	2 (10)	
Total	6 (30)	4 (20)	

Figures in parentheses are in percentages

## DISCUSSION

The combination of newer anaesthetic agents, such as desflurane, remifentanil, sevoflurane and propofol allow us to provide anaesthetic care in an effective and efficient way.[9] When remifentanil infusion 0.3 mg/kg/min is used in patients scheduled for paediatric urologic surgeries, the haemodynamic variables remained stable within their age limits.[10]

Maximum cardiovascular depression can be seen after the first dose of remifentanil and it could be prevented through premedication with glycopyrrolate,[11] and the haemodynamic response to remifentanil appears to be similar to that of other anilidopiperidines.[12] This hypotension and bradycardia associated with remifentanil infusion can be treated with a vasopressor, atropine or a combination of both drugs.[13]

Remifentanil provides haemodynamically more stable anaesthesia compared with ketamine or placebo in paediatric day-care anaesthesia.[14] The mean arterial pressure and heart rate may be decreased in patients receiving remifentanil or propofol.[15]

Patients who received remifentanil showed superior emergence from anaesthesia compared to those who received fentanyl, with significantly shorter time for onset of breathing, eye opening, extubation, and verbalization.[16–18] The time to follow verbal commands and tracheal extubation were more rapid after remifentanil. When compared with the recovery profile of fentanyl, it is reported that remifentanil group shows early recovery.[19]

Cicek[20] compared the effects of propofol–alfentanil or propofol–remifentanil anaesthesia on haemodynamics and recovery characteristics during percutaneous nephrolithotripsy and found that with alfentanil, mean arterial pressure was higher at the first minute in the prone position, and during skin incision and lithotripsy, and heart rate was higher during skin incision and lithotripsy when compared with remifentanil group. The time of recovery for spontaneous ventilation, extubation, and eye opening were significantly shorter with remifentanil than with alfentanil.

In the present study, the time to qualify for recovery-room discharge was significantly shorter in R-group as compared to F-group. Tsui[4] observed the recovery and discharge times of combined remifentanil and propofol total intravenous anaesthesia in spontaneously breathing children and he found the mean recovery and discharge times were 8.9 and 28.2 min, respectively, and one patient experienced post-procedure nausea and vomiting in his study. The recovery characteristics of two anaesthetic techniques in children undergoing short painful oncology procedures, the discharge readiness from the recovery ward was achieved at 19 min after propofol with remifentanil when compared with the combination of propofol, sevoflurane, and nitrous oxide.[21]

Fentanyl provided better PO analgesia during the early PO period than remifentanil; however, there is no significant difference in the number of patients requiring rescue analgesia among two groups. The lower pain scores in the remifentanil anaesthetised patients observed in current study can be attributed to the post-anaesthetic residual effect of fentanyl and the rapid elimination of remifentanil.

The propofol-anaesthetized patients receiving either fentanyl or remifentanil as opioid supplement showed that propofol–fentanyl anaesthesia resulted in a higher incidence of PONV and higher requirements of antiemetic drugs compared with propofol-remifentanil group l.[22] The incidence of PONV after administration of general anaesthesia without antiemetic prophylaxis is reported to be in the range of 35–60%. However, the reduced incidence of PONV reported in the current study can be attributed to pre-operative use of ondansetron and dexamethasone and this reflects the necessity of the use of prophylactic antiemetic when remifentanil is used.

## CONCLUSION

We conclude that remifentanil is appropriate for opioid-based anaesthesia in paediatric patients because it provides haemodynamic stability and rapid recovery characteristics with minimal PO side effects. However, attention must be paid for prophylactic antiemetics and adequate post-operative analgesia.
